# ICTV Virus Taxonomy Profile: *Inoviridae*

**DOI:** 10.1099/jgv.0.001614

**Published:** 2021-07-06

**Authors:** Petar Knezevic, Evelien M. Adriaenssens, ICTV Report Consortium

**Affiliations:** ^1^​ University of Novi Sad, Faculty of Sciences, Department of Biology and Ecology, Trg Dositeja Obradovica 3, 21000 Novi Sad, Vojvodina, Serbia; ^2^​ Quadram Institute Bioscience, Norwich Research Park, Norwich NR4 7UQ, UK

**Keywords:** ICTV Report, taxonomy, *Inoviridae*

## Abstract

Members of the family *Inoviridae* are non-enveloped flexible filamentous bacteriophages (600–2500×6–10 nm) with supercoiled, circular, positive-sense, single-stranded DNA genomes of 5.5–10.6 kb, encoding 7–15 proteins. They absorb to the pili of Gram-negative bacteria and replicate their DNA by a rolling-circle mechanism with progeny released from cells by extrusion without killing the host. Phage DNA can persist extra-chromosomally or integrate into the bacterial genome. This is a summary of the International Committee on Taxonomy of Viruses (ICTV) Report on the family *Inoviridae*, which is available at ictv.global/report/inoviridae.

## Virion

**Fig. 1.**
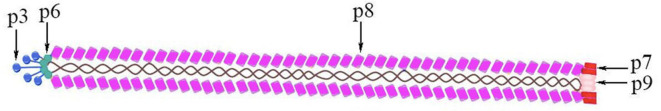
Schematic drawing of Escherichia phage M13 virion.

Virions are non-enveloped, with the major coat protein helically organized around circular, positive-sense, single-stranded DNA [(+) ssDNA], forming long, flexible filaments whose length depends on the genome length, varying from 600 to 2500 nm with a diameter of 6–10 nm (Table 1). Virions of Escherichia phage M13, a member of the species *Escherichia virus M13*, are built up from 2700 copies of the major coat protein (CoaB; p8), with 5 copies each of p7 and p9 forming a blunt end, and of p3 (CoaA) and p6 forming a rounded end (Fig. 1) (reviewed in [1]).

**Table 1.** Characteristics of members of the family *Inoviridae*

Example:	Escherichia phage M13 (V00604), species *Escherichia virus M13,* genus *Inovirus*
Virion	Non-enveloped flexible filaments; 6–10 nm in diameter, 600–2500 nm in length
Genome	5.5–10.6 kb, supercoiled, circular, positive-sense, single-stranded DNA, encoding 7–15 proteins
Replication	Rolling-circle mechanism
Translation	From mRNAs
Host range	Gram-negative bacteria
Taxonomy	Realm *Monodnaviria*, kingdom *Loebvirae*, phylum *Hofneiviricota*, class *Faserviricetes*, order *Tubulavirales*; >20 genera and >25 species

## Genome

**Fig. 2.**
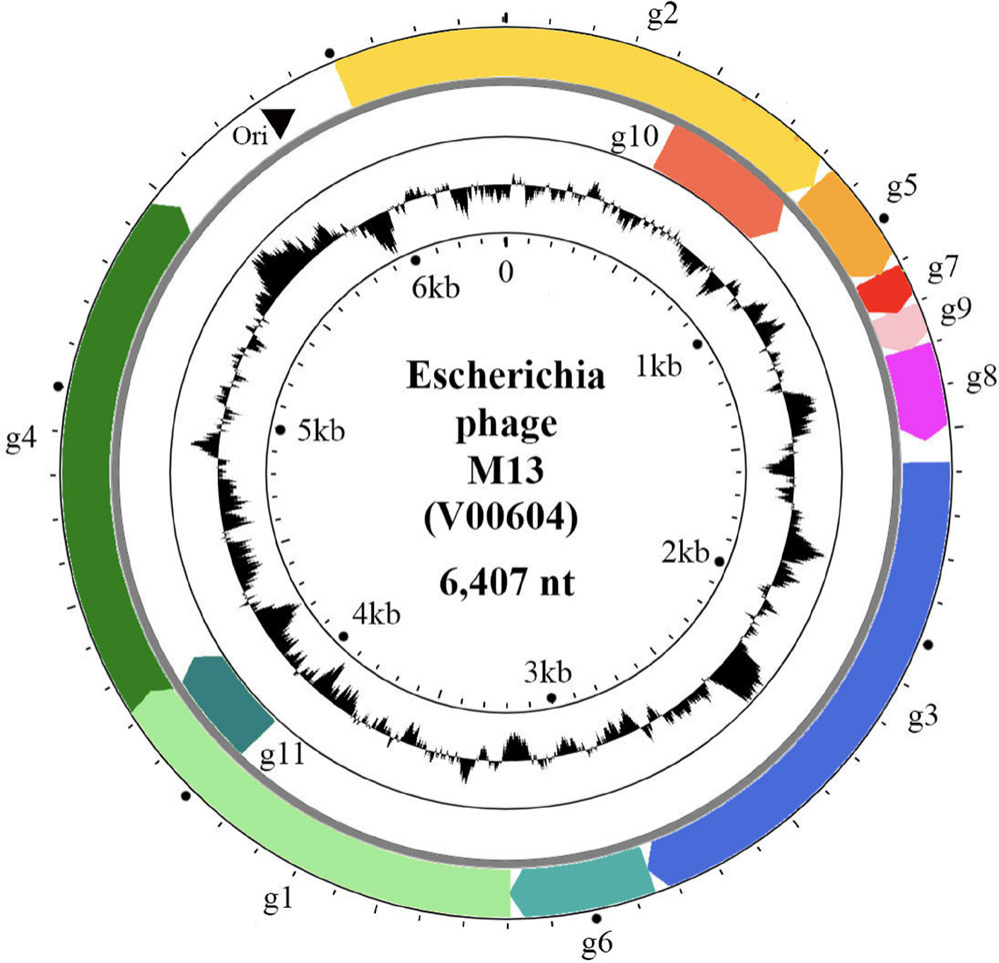
Escherichia phage M13 (V00604) circular genome. Genes (coloured arrows) are organized in modules of replication genes (**g2, g5 and g10**), structural genes (**g7, g9, g8, g3, g6**) and morphogenesis genes (**g1, g4 and g11**). Inner circle indicates GC content.

The circular (+) ssDNA genome of members of the family *Inoviridae* has 7–15 protein coding regions with minimal inter-genic regions; some genes overlap or are embedded within larger genes. The genome is organized in modules (Fig. 2): a DNA replication module, a structural module and a morphogenesis (assembly/extrusion) module, while intergenic regions contain the origin of replication, packaging signals and promoters. Phages that are able to integrate into the bacterial chromosome have genomes containing insertion sequences and encode proteins for latent stage regulation (repressor), while some phages encode integration proteins (integrase or transposase). The repressor genes of integrative phages are usually expressed from the complementary (−) strand. The G+C content is 40.5–60.7 %.

## Replication

For Escherichia phage M13, infection starts when p3 (adhesion protein, CoaA) interacts with pili on the bacterial cell surface (adhesion receptor) and TolQRA co-receptor (entry receptor) [2]. Phage (+) ssDNA is translocated into the bacterial cytoplasm and converted by bacterial polymerases into covalently bound dsDNA, the replicative form (RF) [3]. mRNAs are transcribed from the RF by host RNA polymerases and translated by the host machinery. Replication of DNA starts when p2, with endonuclease activity, nicks dsDNA at the origin site [4]. Host DNA polymerase synthesizes (+) ssDNA from the complementary strand of RF by a rolling-circle mechanism and these are converted back into RFs that serve as templates for transcription. At high concentrations of p5, its homodimers cover newly synthesized (+) ssDNA molecules, preventing further conversion of (+) ssDNA into RF and collapsing (+) ssDNA into filaments. A double-stranded packaging signal remains uncovered at the blunt end of the filament. The structural proteins integrate into the cytoplasmic membrane, together with p4, p1 and p11 that form the assembly machinery [5]. Virions are released by extrusion during the replacement of p5 with p8, the major coat protein (CoaB). Proteins p7 and p9 are the first to extrude, followed by numerous copies of p8, while p3 and p6 are added at the end of the process. New virions are released into the environment, without killing the host.

## Taxonomy

Current taxonomy: www.ictv.global/taxonomy. The family *Inoviridae* belongs to the order *Tubulavirales*, along with the families *Plectroviridae* and *Paulinoviridae*. More than 20 genera are included in the family, many of which only have a single species. Members of the same genus share considerable DNA sequence similarity (identity×query coverage), and also >50 % amino acid sequence similarity for the morphogenesis (Zot; p1) and major coat (CoaB; p8) proteins. Phages belonging to the same species are >95 % identical in DNA sequence over the entire genome length and have significant CoaA amino acid sequence similarity.

## Resources

Full ICTV Report on the family *Inoviridae*: www.ictv.global/report/inoviridae.
